# Effect of Lactylate and *Bacillus subtilis* on Growth Performance, Peripheral Blood Cell Profile, and Gut Microbiota of Nursery Pigs

**DOI:** 10.3390/microorganisms9040803

**Published:** 2021-04-10

**Authors:** Xiaofan Wang, Tsungcheng Tsai, Xiaoyuan Wei, Bin Zuo, Ellen Davis, Tom Rehberger, Samantha Hernandez, Evelien J.M. Jochems, Charles V. Maxwell, Jiangchao Zhao

**Affiliations:** 1 Department of Animal Science, Division of Agriculture, University of Arkansas, Fayetteville, AR 72701, USA; xxw033@uark.edu (X.W.); ttsai@uark.edu (T.T.); xw010@uark.edu (X.W.); binzuo@uark.edu (B.Z.); cmaxwell@uark.edu (C.V.M.); 2 Arm & Hammer Animal and Food Production, Church & Dwight, Inc., Waukesha, WI 53186, USA; Ellen.Davis@churchdwight.com (E.D.); Tom.Rehberger@churchdwight.com (T.R.); Samantha.Hernandez@churchdwight.com (S.H.); 3 Corbion, Arkelsedijk 46, 4206 AC Gorinchem, The Netherlands; e.jochems@corbion.com

**Keywords:** lactylate, *Bacillus subtilis*, growth performance microbiome, pig

## Abstract

To evaluate the effects of lactylate and *Bacillus subtilis* on growth performance, complete blood cell count, and microbial changes, 264 weaning pigs were assigned to four treatments (1) control (Con) basal diets that met the nutrient requirement for each phase, (2) 0.2% lactylate (LA), (3) 0.05% *Bacillus subtilis* strains mixtures (BM), or (4) the combination of LA and BM (LA+BM) added to the control basal diet at their respective inclusion rates in each of the three phases. Dietary lactylate tended to increase weight gain, significantly increased feed intake, and reduced fecal total *E. coli* and enterotoxigenic *E. coli* counts during Phase 1. Pigs fed *Bacillus subtilis* had a greater gain to feed ratio (G:F) during Phases 1 and 2. Pigs fed lactylate had an increased peripheral absolute neutrophil count on D14 but a decreased eosinophil percentage. Pigs fed *Bacillus subtilis* had an elevated peripheral total white blood cell count at study completion. The addition of lactylate increased microbiota richness, reduced *E. coli*, and increased Prevotella, Christensenellaceae, and Succinivibrio. *Bacillus subtilis* supplementation-enriched f_Ruminococcaceae_unclassified and S24-7_ unclassified had positive relationships with feed efficiency. Collectively, these findings suggested that lactylate can be added to diets to balance gut microbiota and improve growth performance during the early postweaning period. The combination of lactylate and *Bacillus subtilis* strains exerted a synergic effect on the growth performance of nursery pigs.

## 1. Introduction

Postweaning is the most challenging period of life for swine because environmental changes and diet formulations force the reprogramming of the digestive system and the gut microbiota community [1,2]. Under this circumstance, the ecological balance of gut microbiota can be severely disturbed, and piglets can become very susceptible to pathogens that cause diarrhea, morbidity, and mortality [3]. Health during the early nursery period has a long-term impact on growth rates and feed efficiency, all of which have significant economic consequences for swine producers [4]. Maintaining gut homeostasis is considered the primary means to mitigate the detriments associated with weaning. Most strategies for addressing weaning stressors are gut-microbiota-associated [5,6]. This is because the gut microbiota have a multitude of functions in host defense, such as nutrient metabolism, energy replenishment, and establishing gastrointestinal barrier integrity [7,8]. 

Dietary energy contributes to the greatest proportion of feed cost in the swine industry [9,10]. In addition to carbohydrates and fat from grain, dietary fat and oils are major energy sources. Fat digestion begins with emulsification by bile to form micelles, which increases the accessibility to pancreatic lipase [11]. The digestive system of weaning piglets is incompetent in processing dietary fat from solid diets [12]; hence, supplementing exogenous emulsifiers can promote fat absorption and retention. Lactylates (LA) are esterified lactic acid and fatty acid compounds that have been approved by the Food and Drug Administration for use as emulsifiers in food production [13]. Recently, growing evidence of its benefits on animal husbandry has been described [14]. Several studies have demonstrated the beneficial effects of sodium stearoyl-2-lactylate (SSL) on growth performance and nutrient digestibility in young pigs and producing sows [15,16]. Similar responses were also observed in broilers when SSL (0.05–0.1%) was supplemented in a common diet [17] or an energy-reduced diet [18].

Direct-fed microbiota (DFM) are beneficial microorganisms that are commonly used in diet interventions to improve livestock health. There are three major types of DFM in the swine industry: Lactobacillus, yeast, and Bacillus. Certain *Bacillus* sp., the most preferred DFM, are spore-forming aerobes [19] with thermostable and low-pH-resistant characteristics. They can be easily mixed into solid feed and survive the digestive system. These *Bacillus* sp. have been determined to possess beneficial probiotic capabilities on growth performance and disease resilience in pigs via gut microbiota manipulation [20]. For instance, Hu et al. found that a *Bacillus KN-42*-supplemented diet not only improved growth performance but also reduced the incidence of diarrhea in weaned pigs, perhaps by stimulating *Lactobacillus and suppressing E. coli* [20,21]. Other research institutes unveiled the defensive mechanisms of beneficial Bacillus against enterotoxigenic *E. coli* (ETEC) in young piglets by noting that piglets that received Bacillus probiotics had enhanced goblet cell function and gut integrity, which ameliorated ETEC-induced enteritis [22,23].

The purpose of the current project is to examine the effects of active lactylate (LA) and *Bacillus subtilis* strain mixtures (BM) on growth performance, peripheral blood cell counts, pathogen count, and the gut microbiome in pigs. By profiling the gut microbiota in each treatment, we discuss the potential mechanisms of how the driven microbiota community influences the growth performance of nursery pigs and use a machine learning method to reveal taxa that best represent the impact of the treatments.

## 2. Materials and Methods

Pigs were managed according to the University of Arkansas Institutional Animal Care and Use Committee guideline (IACUC# 21038).

### 2.1. Animals, Experimental Design, and Dietary Treatments

A total of 264 weanling pigs (PIC1050 × DNA600) were selected at weaning (Day 21 ± 2) and transferred to a conventional nursery facility at the University of Arkansas Swine Research Unit. Pigs were blocked by initial body weight into 11 groups within each sex and allotted to 1 of 44 plastic floor pens. Pens then were randomly assigned to one of four dietary treatments in a 2 × 2 factorial arrangement, including (1) control (Con) basal diets that met the nutrient requirement for each phase (Table S1), (2) 0.2% lactylate (LA), (3) 0.05% *Bacillus subtilis* strains mixtures (BM), or (4) the combination of LA and BM (LA+BM), added to the control basal diet at their respective inclusion rates in each of the three phases. The types of active lactylate (ALOAPUR^®^, Corbion, The Netherlands) used in this trial were ester compounds made from lactic acid with lauric acid and myristic acid, using diatomaceous earth material as the carrier. *Bacillus subtilis* strains (B. 747 + B. 1999) were supplied by Arm and Hammer Animal and Food Production (Certillus™, Waukesha, WI, USA). All diets were devoid of feed antibiotics and pharmaceutical levels of zinc and copper.

Each pen housed three male and three female pigs, and they remained on the same treatment throughout the study period. Pigs were fed a three-phase dietary regimen with a 14-day duration per phase (Phase 1: D0–14; Phase 2: D14–28; Phase 3: D28–42). Each pen (size: 1.5 × 1.2 m^2^) was equipped with a two-hole feeder and a cup waterer, and pigs had free access to feed and water during the entire trial. The nursery facility was a totally confined building, and the ambient temperature was programmed to start at 29 °C at weaning and declined sequentially to 25 °C by the end of the trial. Fluorescent lighting was provided 24 h per day during the entire study.

### 2.2. Growth Performance, Sample Collections, and Processing

Individual pig body weight (BW) was measured at weaning and the end of each phase to calculate average daily gain (ADG). Feed intake was recorded throughout the trial to determine the average daily feed intake (ADFI) during each phase. Feed efficiency (FE or G:F) was defined as the amount of body weight gain from a given pen divided by the feed consumed during a period. Body weight, feed added, and leftover feed was weighed using a customized weight scale (IQ Plus^®^390DC, Rice Lake Weighing System, Rice Lake, WI, USA). A 50-lbs weight block was used to validate the reading of the scale prior to and post weighing. Fresh feces, rectal swabs, and blood samples were collected from a median body weight barrow in each pen at weaning, and the same pigs were sampled during the entire trial.

Fecal samples were assayed for Clostridium, *E. coli*, and *Streptococcus suis* counts at the Arm and Hammer Animal and Food Production Laboratory (Waukesha, WI. USA), following procedures outlined in the Bacteriological Analytical Manual (8th Ed., 1998). Briefly, fecal samples were diluted 1:10 in 0.1% peptone, masticated, and plated onto selective media. Total Clostridium and *C. perfringens* were isolated using Perfringens agar base (Oxoid Ltd., Cambridge, UK) with tryptose sulfite cycloserine (TSC) supplement (Oxoid Ltd.). *Clostridium perfringens* isolates were confirmed using multiplex PCR for the detection of alpha toxin [24]. *Escherichia coli* were enumerated on CHROMagar^TM^ (CHROMagar, Paris, France). Isolates were confirmed as enterotoxigenic *E. coli* (ETEC) using multiplex PCR methods adapted from Stacy-Philips et al. [25] and Casey and Bosworth [26]. Streptococcus was isolated and enumerated on Todd–Hewitt broth (THB), and *S. suis* was confirmed by PCR using the primer set developed by Okwumabua et al. [27].

Plasma lactylates were analyzed via liquid chromatography using an Acquity UPLC system (Waters, Milford, MA, USA) with an analytical column (Acquity UPLC BEH C18, 50 × 2.1 mm i.d., dp = 1.7 µm (Waters)) and an Inline filter (ASSY frit, 2.1. mm id., 0.2 µm (Waters)) at a column oven temperature of 40 ± 1 °C.

The rectal swabs were frozen immediately at –80 °C until microbiome analysis. Blood samples were drawn via jugular vena and collected into K2EDTA-coated vacutainer tubes for plasma lactylate evaluation (Charles River Laboratories, ‘s-Hertogenbosch, The Netherlands) and complete blood count (CBC) determination using a Hemavet 950 (Drew Scientific, Miami Lakes, FL, USA).

### 2.3. 16S rDNA Amplicon Sequencing

A volume of 200 μL of fecal suspension from each rectal swab (*n* = 172) was used for 16S rDNA sequencing, as previously described [28]. Briefly, DNA extractions were performed with the PowerLyzer PowerSoil DNA Isolation Kit (Qiagen, Germantown, MD, USA), following the manufacturer’s protocol. Extracted DNA was subjected to quantity and quality determination by a NanoDrop spectrophotometer (Thermo Fisher Scientific, Wilmington, DE, USA), followed by library construction and 16S rDNA amplicon sequencing. Bacterial 16S rDNA V4 amplicons were generated by polymerase chain reaction (PCR) using primers (F: 5′- GTGCCAGCMGCCGCGGTAA -3′ and R: 5′- GGACTACHVGGGTWTCTAAT -3′) with the attachment of eight base pairs of the barcoded index and Illumina flow cell adapters. Accuprime Pfx Supermix (Invitrogen, Carlsbad, CA, USA) was used as the PCR reagent because of its high fidelity amplification. PCR products were cleaned and normalized by a SequalPrep Normalization Plate Kit (Invitrogen, Carlsbad, CA, USA) to balance DNA densities between samples. A Qubit 3 fluorometer (Thermo Fisher Scientific, Waltham, MA, USA) was used to validate the normalization step, and then purified DNAs were pooled in equal volume. The DNA pool was further subjected to quality and quantity determination with an Agilent Bioanalyzer 2100 (Agilent, Santa Clara, CA, USA) and quantitative RT-PCR, respectively. For sequencing, Illumina MiSeq 2 × 250-bp paired-end sequencing (MiSeq Reagent Kit v2, 500 cycles, 20% PhiX) was used. Before loading into the cartridge, DNA pools were denatured with freshly made 0.2 N NaOH solution and then diluted with HT1 buffer. NaOH-denatured Phix was incorporated into the final pool at 20% to increase nucleotide diversity at each cycle to improve the reading. Negative control and mock communities (ZymoBIOMICS™ Microbial Community Standard; Zymo, Irvine, CA, USA) were included as two samples for future quality evaluation.

### 2.4. Statistical Analysis for Growth Performance, Complete Blood Cell Count, and Microbial Counts

Growth performance measures (BW, ADG, ADFI, and G:F) and complete blood cell count (CBC) data were analyzed by ANOVA using the mixed procedure of SAS 9.3 (SAS Institute, Inc., Cary, NC, USA) in a randomized complete block design, with a 2 × 2 factorial dietary treatment arrangement. The pens served as the experimental unit. The model included a block as the random effect, with a 0.2% lactylate and 0.05% *Bacillus subtilis* mixture and its interaction as the fixed effects. Student’s *t*-test was used in multiple comparisons to test the significance. The microbial count data were analyzed by diet phase using the GLM procedure of JMP^®^ version 15.0.0 (SAS Institute, Inc., Cary, NC, USA, 1989–2019). A *p*-value <0.05 was considered significant, and 0.05 < *p*-value < 0.10 was considered a statistical trend.

### 2.5. Microbiota Data Analysis

Raw reads (forward and reverse for each sample) generated by Miseq were uploaded into the QIIME2 platform (v. 2.4). Sequencing reads analysis followed general steps, including paired reads joining, quality filtering, denoising (Deblur in QIIME2), classification (Greengenes reference database; version 13-8), rarefying (5982), and bacterial units clustering (100%). Alpha (Shannon index, observed features) and beta diversity (Bray–Curtis) were evaluated for microbial diversity and distances between subjects, respectively. Differentially represented bacterial members between groups were determined using the online software LEfSe (https://huttenhower.sph.harvard.edu/galaxy/; access date: 1 December 2020). Classification and regression-based random forest models were developed to identify treatment- or growth-performance-associated bacterial members, respectively. Mothur software v. 1.39.5 (corr.axes) was used to calculate the coordinates for each significantly affected blood component. R software was used for data visualization.

This sequencing and data analysis pipeline yielded high-quality reads, as suggested by the clustered mock communities’ consistent relative abundances of each species between different Miseq runs.

## 3. Results

No significant clinical symptoms of diseases were observed throughout the study except for minor postweaning scours. Scours were not associated with any of the treatments. Two pigs, one each from Treatments 2 and 4, were removed from the trial due to illness. Plasma C12 and C14 lactylate measurements demonstrated that the active lactylates used in this trial were absorbed from the lumen of the GI tract and translocated into systemic circulation. As shown in Table S2, the plasma concentrations of both C12 and C14 lactylates were below the detectable limit (<5 ng/mL) in control pigs, while the levels of C12 and C14 lactylates were detected at around 125 and 15 ng/mL, respectively, in pigs fed a diet supplemented with lactylates throughout the study (Table S2). In addition, plasma C12 and C14 lactylates in pigs fed 0.2% LA were higher at the end of Phases 2 and 3 than Phase 1. However, it gradually declined from Phases 1 to 3 in pigs fed both LA and BM.

### 3.1. Growth Performance, Complete Blood Count, and Microbial Counts

**Figure 1:**
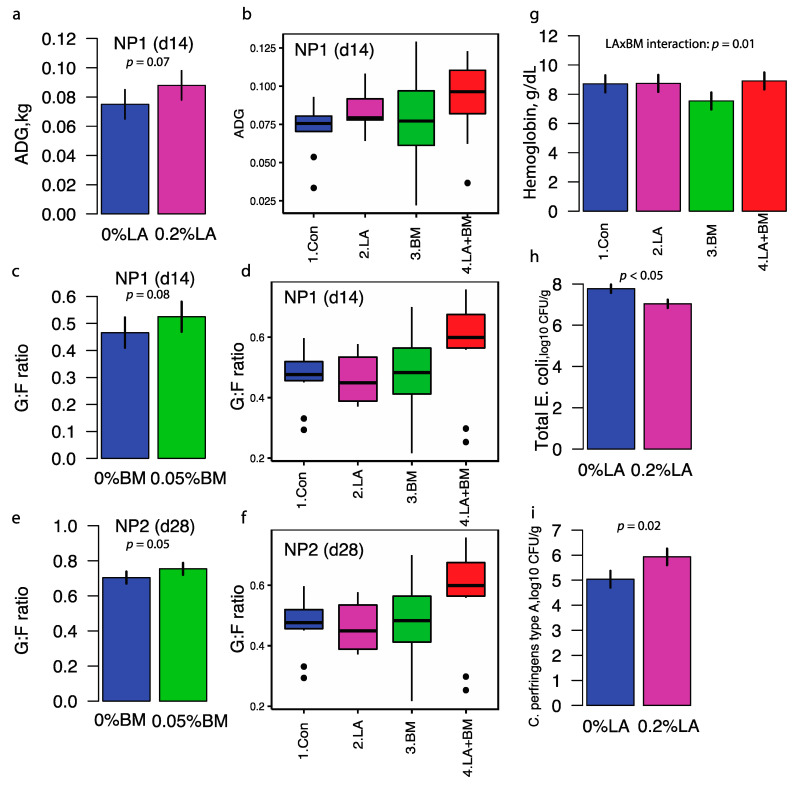
Effects of lactylate (LA) and *Bacillus subtilis* (BM) on average daily gain (ADG) at the end of nursery phase 1 (NP1) (**a**,**b**); feed efficiency at the end of NP1 (**c**,**d**) and NP2 (**e**,**f**); hemoglobin (**g**); *E. coli* counts at the end of NP1 (**h**); *C. perfringens* type A counts at the end of NP3 (**i**). A total of 264 pigs of weaning age were stratified by initial body weight (BW) and gender and assigned to a 2 × 2 factorial arrangement trial. The four dietary treatments were (1) control (Con), (2) 0.2% lactylate (LA), (3) 0.05% *Bacillus subtilis* strain mixtures (BM), or (4) the combination of LA and BM (LA + BM), added to the control basal diet at their respective inclusion rates in Treatments 2 and 3. All diets were devoid of feed antibiotics and pharmaceutical levels of zinc and copper. Pigs were fed a three-phase regimen, with a 14-day duration per phase. Pigs’ body weight, feed intake, and fecal samples were collected at the end of each phase. Blood samples were collected to determine plasma lactylates and complete blood cell count.

The addition of lactylates to the diet tended to increase average daily gain (ADG) compared to pigs fed diets devoid of lactylates (0.088 vs. 0.075 kg/d; LA: *p* = 0.07; Table S3 and Figure 1a,b). No statistically significant differences in ADG were observed in pigs fed the *Bacillus subtilis* mixture. Pigs fed diets containing lactylates alone had an increased average daily feed intake (ADFI) during Phase 1 but not when fed in combination with BM (Table S4, LA × BM interaction *p* = 0.03). Pigs fed the *Bacillus subtilis* mixture tended to have a greater gain-to-feed ratio (G:F) during Phase 1 (BM: *p* = 0.08; Figure 1c,d) and Phase 2 (BM: *p* = 0.05; Figure 1e,f) than pigs fed the *Bacillus-subtilis*-free diets. The improvement in G:F in pigs fed *Bacillus subtilis* was mainly driven by pigs fed diets supplemented with both lactylate and *Bacillus subtilis* mixtures (Phases 1 and 2, LA x BM interaction, *p* = 0.04; Table S4). Although there were no significant treatment effects on body weight (*p* > 0.10 for all three phases), pigs fed diets containing both lactylates and *Bacillus subtilis* were 0.26, 0.63, and 0.85 kg heavier than the control groups at the end of Phases 1, 2 and 3, respectively (Table S4).

Both dietary lactylates and the *Bacillus subtilis* mixtures caused noticeable effects on peripheral blood cell counts. Pigs fed lactylates had a higher absolute neutrophil (NE) count at the end of Phase 1 (D14) compared to pigs fed diets devoid of LA; however, the NE count decreased over time to values lower in pigs fed LA on D28 and D42 than in pigs fed diets devoid of LA (Table S5, LA×day *p* = 0.04). The percentage of the eosinophil in peripheral blood decreased at a greater magnitude in pigs fed with LA compared to their counterparts on D14. The percentage of monocytes over total white blood cell count increased over time from the end of Phase 1 to study completion in pigs fed LA, whereas there was a lack of time response in pigs fed LA-free diets (Table S5, LA×day *p* < 0.01). An age-associated response in peripheral total white blood cell counts differentiated BM from BM-free diets. Absolute total white blood cell counts increased from weaning to the end of Phase 2 (d28) in both pigs fed BM and BM-free diets. While the level of peripheral total white blood cells remained similar in pigs fed BM at the end of the trial, a decline was observed in pigs fed BM-free diets (Table S6, BM×day *p* < 0.01). This observation on total white blood cell counts was mainly driven by absolute lymphocyte count (Table S6, BM×day *p* < 0.01). An expansion of absolute neutrophil count occurred in both BM and BM-free treatments from Phases 1 to 2. However, the magnitude of expansion was lower in BM than BM-free treatments (Table S6, BM×day *p* = 0.06). Both red blood cell counts and the percentage of hematocrits decreased from weaning to the end of Phase 2 in pigs fed BM-free diets, but the levels were relatively stable in pigs fed BM-supplemented diets across age (Table S6, BM×day *p* = 0.07). Pigs fed BM had the lowest hemoglobin concentrations, whereas the diets containing lactylate alone and in combination with BM had similar hemoglobin levels compared to the control pigs (LA×BM interaction, *p* = 0.02; Figure 1g).

Dietary lactylates reduced the bacterial cell counts of major pathogens during the early nursery stage, which coincided with weight gain improvement. At the beginning of the study, the *Streptococcus suis* cell count in feces in pigs fed only LA was similar to those pigs fed control diets. While pigs fed BM alone had similar counts of *Strep suis*, a lower count was showed in pigs assigned to the combination of BM and LA (BM×LA interaction, *p* = 0.05), but this pattern disappeared after that (Table S7). Supplementing LA in the diet reduced (*p* < 0.05) fecal total *E. coli* (6.62 ± 0.29 vs. 7.72 ± 0.30 log_10_ CFU/g; *p* = 0.01) and enterotoxigenic *E. coli* (6.62 ± 0.30 vs. 7.72 ± 0.29 log_10_ CFU/g; *p* = 0.01) counts at the end of Phase 1 compared to pigs fed diets devoid of LA (Figure 1h). At the end of Phase 3, pigs fed diets containing LA had greater (*p* = 0.02) total Clostridium (6.31 ± 0.23 vs. 5.11 ± 0.23 log_10_ CFU/g; LA: *p* < 0.01) and *C. perfringens* type A (5.93 ± 0.27 vs. 5.04 ± 0.26 log_10_ CFU/g; LA: *p* = 0.02; Figure 1i) presence in feces compared to pigs fed diets without LA.

### 3.2. Microbial Composition in Nursery Pigs

**Figure 2:**
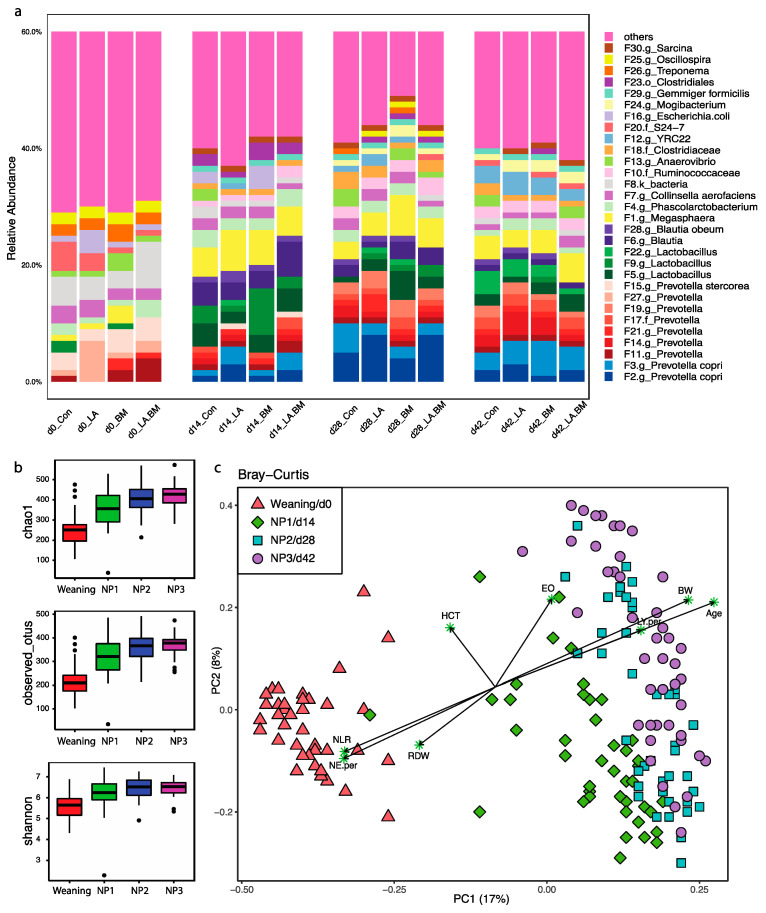
Characterizations of gut microbiome compositions (**a**) and community dynamics (**b**) and alpha diversity and (**c**) beta diversity at weaning (d0) and the end of each nursery phase (NP): NP1 (d14), NP2 (d28), and NP3 (d42). Rectal swabs were collected from all four treatments at the end of each phase and subjected to DNA extraction and 16S rDNA amplicon sequencing. Mothur software v.1.39.5 was used to calculate the coordinates for each significantly affected blood/bacterial component (EO, eosinophil; HCT, hematocrit; NLR, neutrophil–lymphocyte ratio; RDW, red cell distribution width; LY.per, percentage of lymphocyte).

A total of 172 rectal swabs passed the quality and rarefying procedures, yielding a total of 1,028,904 high-quality reads clustered into 2438 features. These features were classified into 20 phyla and 182 genera (Figure 2a; Figure S1). Throughout the entire nursery stage, Firmicutes, Bacteroidetes, and Proteobacteria consistently represented the top three most dominant phyla, contributing 52%, 30%, and 3.8% to overall phyla, respectively. There is an obvious transit of the microbial community at the genus level from weaning to nursery pigs. *Prevotella* is the most dominant genus member at weaning, and the dominance continues to increase during the nursery phase, maintaining the highest abundance among all taxa. Similar to Prevotella, genera Lactobacillus, Ruminococcaceae, Megasphaera, and Blautia increased rapidly in the first two weeks after weaning, emerging as other dominant members in the nursery phase (Figure S1). Some taxa such as Bacteroides, Fusobacterium, and Mogibacterium, occupying 1.8~6% of total taxa at weaning, were adversely affected regarding their abundances (<0.005%) at the end of Phase 1 (Figure S1). A similar pattern was observed at the feature level; nine Prevotella features and three Lactobacillus features were enriched greatly on the first observation day, although to a different extent (Figure 2a).

### 3.3. Effect of Age on Gut Microbial Structures and CBC Profiles

Microbiota diversity and richness steadily increased throughout the nursery stage, as indicated by the increasing alpha indexes of Chao1, observed features, and Shannon (Figure 2b). During the first two weeks, the communities demonstrated the greatest diversity change among all three time intervals, after which the change slowed until the end of the study. As shown in the principal coordinate analysis (PCoA) plot based on Bray–Curtis distances, community structures of pigs on weaning day were significantly different from those of older nursery pigs (Figure 2c). However, despite being distinguishable, Phase 1, Phase 2, and Phase 3 microbiota communities had greater similarities than differences when compared to weaning day observations. These observations were in line with both alpha diversity and compositional changes during the crucial transition from a sow milk diet to solid feed (Figure 2c). By aligning the Bray–Curtis-distance-based coordinates with significantly changed blood cell counts and phenotypes, neutrophil–lymphocyte ratio (NLR), NE%, and red cell distribution width (RDW) were more correlated with gut microbiomes in weaning pigs fed sow milk, while EO and lymphocyte percentages were more associated with those in nursery pigs fed a solid diet. This relationship was enhanced in the older-age nursery pigs (Figure 2c).

**Figure 3:**
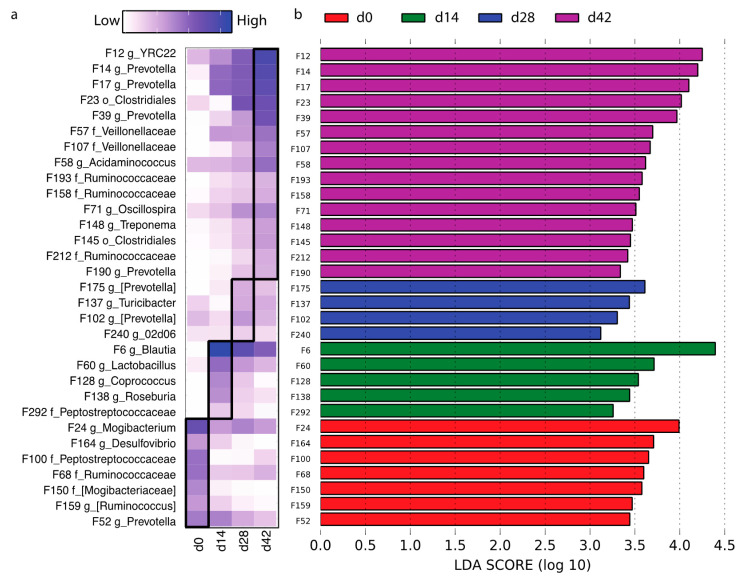
Abundances of age-associated bacterial features at weaning (d0) and the end of each nursery phase: d14, d28, and d42 (**a**). Discrepant bacterial members between groups were determined using online software LEfSe (**b**) (https://huttenhower.sph.harvard.edu/galaxy/; access date: 1 December 2020).

To identify the different bacterial features within the four time points, feature tables (top 300 features) from all samples were included for linear discriminant analysis effect size (LEfSe, Figure 3a,b). A total of 31 features were detected to be day-associated. Specifically, F24 *Mogibacterium*, F164 *Desulfovibrio*, F150 *Mogibacteriaceae_unclassified*, and F52 *Prevotella* tended to be highly presented in weaning pigs. Five features, including F6 *Blautia*, F60 *Lactobacillus*, and F128 *Coprococcus,* were enriched at the end of NP1 (D14). Four features (F175/F102 *Prevotella*, F137 *Turicibacter*, and F240 02d06) were NP2-associated (D28). At the end of NP3, when the pigs were two months old, a greater number of microbial features were discovered at a higher abundance, which included four *Prevotella* (F14, F17, F39, and F190), three *Ruminococcaceae_unclassified* (F193, F158, and F212), and two *Veillonellaceae_unclassified* (F57 and F107; Figure 3a,b).

### 3.4. Effects of Dietary Lactylate and Bacillus Subtilis on the Microbiota Community in Nursery Pigs

Lactylate and *Bacillus subtilis* had a crucial impact on growth performance. Diet is one of the main drivers of gut microbiome structure. Studying the altered microbial taxa in the gut can provide a better understanding of the principles leading to beneficial effects. In this section, we investigated how dietary supplements (lactylates or *Bacillus subtilis*) modulated overall community structure and individual bacterial taxa members.

**Figure 4:**
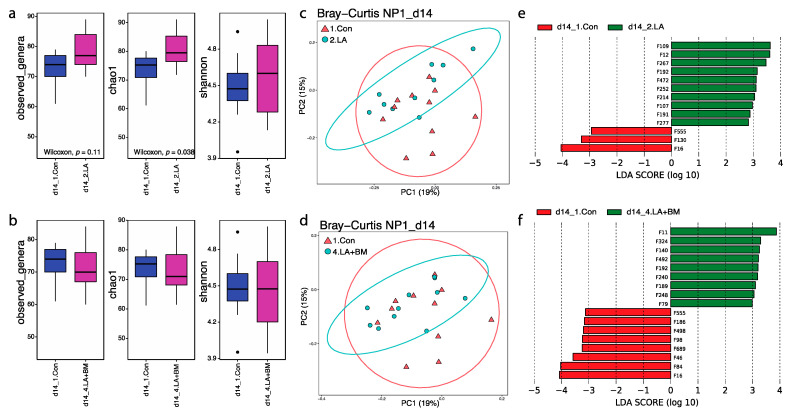
Dietary lactylate (2.LA and 4. LA+BM) modulated gut microbiota (alpha diversities: (**a**,**b)**; beta diversities: (**c**,**d**)) and single bacterial members (**e**,**f**) during the first phase after weaning. Dietary lactylate increased microbial richness, and Bray–Curtis distances show a distinguished separation between the two groups. LEfSe disclosed the features with significant changes when adding lactylate to the diet.

Pigs that received dietary lactylate (Treatment 2 (2.LA) and Treatment 4 (4.LA + BM)) had improved ADG at Phase 1 (D14; Figure 1b). Next, we studied how the treatments affected their microbiota community shifts. Generally, adding lactylate (2.LA) in the diet increased richness at the genus level when compared to pigs fed control diets, as indicated by observed genus and Chao1 (Figure 4a). No significant change was observed for community diversity (Shannon) between the 1.Con and 2.LA groups. Interestingly, none of the alpha indexes were affected by supplementing both lactylates and *Bacillus subtilis* in the diet (4.LA+BM; Figure 4b). Beta-diversity based on Bray–Curtis dissimilarities presented distinguishable separations for both comparisons: 1.Con vs. 2.LA and 1.Con vs. 4.LA+BM (Figure 4c,d). To further reveal the altered bacterial members by treatments, the top 300 bacterial features from both groups were used for LEfSe analysis. In the comparison between 1.Con and 2.LA, only three bacterial members (F16 *E. coli*, F130 Eubacterium, and F555 Ruminococcaceae) were enriched in the control group, while ten members (such as F12 YRC22, F267 Christensenellaceae, F191 Succinivibrio, and F192 Clostridials_unclassified) were exclusively higher in abundance in the 2.LA treatment (Figure 4e). When comparing 4.LA+BM with the 1.Con groups, the relative abundances of F11 Prevotella, F192 Clostridials_unclassified, F189 Coriobacteriaceae, F79 Treponema, F492 Ruminococcaceae, and F248 Bacteroidales increased by the additional supplementation of lactylates and Bacillus subtilis, while F555 Ruminococcaceae, F16 E. coli, F46 Roseburia faecis, and F84 [Prevotella] had decreased relative abundance (Figure 4f). Overall, F16, F555, and F192 revealed a similar pattern in both comparative groups. These changes were possibly driven by dietary lactylate. It is worthy to note that the most abundant *E. coli* (F16) was consistently present in weanling pigs of all groups, from 1% to 4%, while adding lactylate in the diet reduced the amount of F16 *E. coli* to under the minimum detectable level at the end of Phase 1: 1.Con—2%, 2.LA—0%, 3.BM—4%, and 4.LA+BM—0% (Figure 2a).

We continued to analyze how feed-efficiency-associated *Bacillus subtilis* modulated microbial structures at the end of Phase 2 (D28). Similar or slightly reduced alpha diversities (observed_genus, Chao1, and Shannon) were observed in 2.BM and 4.LA+BM groups compared with the Con group separately (Figure S2a,b). PCoA based on Bray–Curtis distances disclosed differences in microbiota structures between BM or BM-devoid diets (Figure S2c,d). Bacterial features such as F1 Megasphaera, F112 Coprococcus, F189 Coriobacteriaceae_unclassified, F207 Gemmiger, and F326 [Prevotella] increased in pigs fed a diet supplemented with only BM (3.BM), while relative abundances of F564 Ruminococcus, F171 Dialister, F207 Gemmiger, F143 Succinivibrio, and certain others decreased (Figure S2e). Adding both LA + BM to the nursery diet increased F29 *Gemmiger formicilis*, F112 Coprococcus, F39 Prevotella, F591 Mitsuokella, and F395/F410 Ruminococcaceae in the gut at NP2 but decreased the abundances of F135 Prevotella, F584 Bacteroides, F143 Succinivibrio, F34 Prevotella, and F20 *S24-7* (Figure S2f). Overall, the *Bacillus subtilis* treatment enriched F112 and reduced F20 and F143 in pigs at the end of NP2.

### 3.5. Feed Efficiency and ADG-Associated Bacterial Features

**Figure 5:**
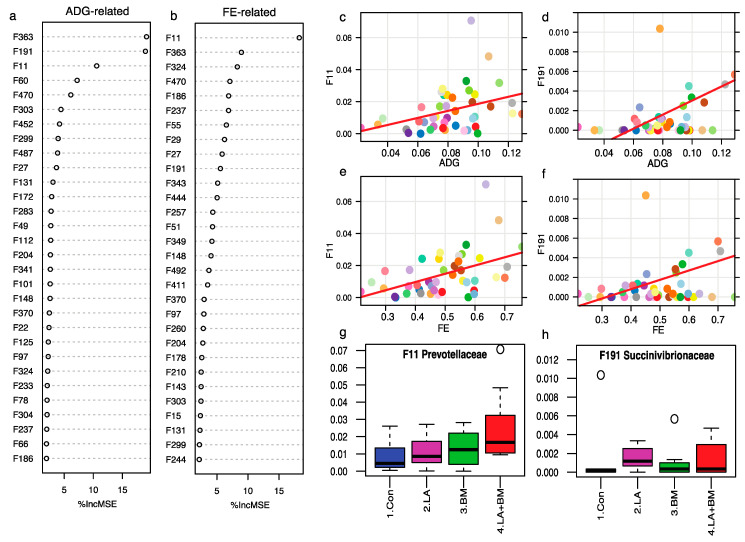
Regression-based random forest disclosed ADG-(**a**) and feed efficiency (FE)-associated (**b**) features (Top 30) at the end of nursery phase 1 (NP1). Bacterial features F11 and F191 were positively related to ADG (**c**,**e**, respectively) and FE (**d**,**f**, respectively). Additional lactylates in the diet can enrich both features at NP1 (**g**,**h**, respectively).

We observed improved feed intake and average daily gain in pigs fed lactylates during Phase 1 and improved feed efficiency in pigs fed the *Bacillus subtilis* mixture during Phases 1 and 2. These phenotypes were likely enhanced by gut microbiota compositions due to their main roles in fiber digestion and essential nutrient production. We next constructed a regression-based random forest on these observations with the top 400 bacterial features to identify ADG- or FE-associated beneficial taxa. At the end of NP1, F363, F191, F11, F60, and so forth were associated with ADG, while F11, F363, F191, F470, F186 (Figure 5a), and so forth were associated with FE (Figure 5b). Among these features, we observed that F11 Prevotella (Figure 5c,e) and F191 Succinivibrio (Figure 5d,f) presented positive correlations with both ADG and FE. Moreover, F191 increased when adding lactylates (Figure 5h), and F11 increased in diets supplemented with *Bacillus subtilis* or lactylates alone, with diets containing both BM and LA having the highest level (Figure 5g). At the end of NP2, F390, F9, F196, F269, F480, F290, and certain others were the top-most FE-associated taxa; F198 and F141 possessed positive relationships with FE, and both were enriched by the *Bacillus subtilis* treatment (Figure S3).

## 4. Discussion

### 4.1. Lactylate and Bacillus subtilis Effects on Phenotypes

Dietary intervention has become a very important focus within the swine industry to help minimize the detrimental effects of weaning [10,19,29,30]. The current study characterized the capabilities of probiotics and lactylates on growth performance, feed efficiency, peripheral blood cell counts, and gut microbiota changes during the nursery stage.

Piglets fed a lactylate-supplemented diet containing esters of lactic acid and various medium-chain fatty acid (lauric and myristic acid) compounds resulted in increased ADG and ADFI in the first two weeks postweaning. Our results are in line with previously published studies where piglets fed with 0.1% sodium stearoyl-2-lactylate in the diet significantly increased ADG and ADFI during the first 17 days [15]. An increased feed intake postweaning indicates a healthier gut environment physically and histologically. However, no significant growth-promoting effects were observed for the remaining trial period, which was similar to our results: a significantly improved ADG was only observed during the first two weeks of lactylate application. Hence, lactylates could be supplemented in the early postweaning diet as transient pacifiers to minimize weaning stress and to reduce the incidences of disease, difficulty of management, and economic cost. In our study, *Bacillus subtilis*, a mixture of two *Bacillus subtilis* strains, significantly increased feed efficiency during the early nursery stage. Interestingly, there was a synergistic improvement in feed efficiency when lactylate and *Bacillus subtilis* were both added to the diet during the first four weeks. The feed conversion ratio is one of the major parameters assessing economic value for the swine industry; thus, *Bacillus subtilis* could represent a feed efficiency booster and have potential benefits to growth performance, gut health, tight junctions, immune response, and the gut microbiome [20,21,22,23].

The circulatory system is the carrier for the cycling of oxygen, nutrients, wastes, and other functional molecules [31]. It also assists the immune system by transporting cells between the central and peripheral lymphoid organs. Eosinophils are immune-related white blood cells that can help defend the host by releasing toxic compounds against invading pathogens [32]. It has been reported that an increase in circulating eosinophils is associated with stimulated intestinal tissue repair function when infection occurs [33,34]. In contrast, there was a greater decline of peripheral eosinophil percentage in pigs fed lactylate at the end of Phase 1, while other phases remained consistent. This potentially indicated that those pigs were situated under a healthier status compared to their counterparts. A similar pattern was observed in feed intake and enterotoxigenic *E.coli* count. Caloric intake is suggested to promote the homing of eosinophils to small intestine tissue via the activation of type II innate lymphoid cells [35]. Therefore, higher feed intake in Phase 1 could be the cause of the declining percentage of eosinophil in pigs fed supplemented lactylate. Moreover, the reduction in ETEC count indicates a lower risk of infection, and increased intake contributes to improved growth performance rather than tissue repairing. To further explain the relationships with gut integrity, measures of eosinophils and tight junction proteins in the local tissues are required. Hemoglobin counts and hematocrit% are parameters of risk for anemia in weaning pigs. Piglets with a hemoglobin concentration lower than 9.0 g/dL are classified as anemic [36]. Hence, lactylate could reduce the incidence of anemia in weaned pigs by increasing hemoglobin and hematocrit% [37].

One cause of high morbidity and mortality in postweaning pigs is their high susceptibility to pathogens. We quantified the populations of *E. coli* and ETEC in their feces by the spread plate technique and observed a significant reduction in their numbers, which correlated with the addition of dietary lactylate. This suggested that lactylate could be a potential replacement for antibiotics to help protect weaned pigs by reducing pathogen abundance.

### 4.2. Longitudinal Gut Microbiome Dynamics

We utilized the 16S rDNA amplicon sequencing technique and observed gut microbiome transformations from weaning to the end of the nursery stage, which involved two very different living conditions. The 20-day-old piglets had to endure substantial stressors mainly from two aspects: (1) from an easily digestible sow milk diet to less-digestible solid feed and (2) from a sow/litter isolated facility to a crowded nursery where pen mates are a mix of different litters. The abrupt physical and dietary changes, generating considerable stress, can cause digestion reconstruction and immune system disruption, which can lead to impaired gut integrity and dysbiosis [7]. Correspondingly, the gut microbiota community undergoes reestablishment by peer competition and environmental selection. From weaning to NP1, there were significant differences in microbiota composition due to various available fiber sources and other undigested compounds in the distal gut. For instance, *Prevotella copri* was observed to develop rapidly during this period due to the increased plant fiber in the diet [38]. Their relative abundance increased during NP2 and then slightly decreased in NP3. Lactobacillus (F5, F9, and F22), Blautia (F6 and F28), and Megasphaera (F1) were three taxa that multiplied considerably during NP1; they are known to consume dietary fiber [39]. In addition, various appearing preferences were also discovered for bacteria under the same genus classification: Prevotella (F15 and F27) increased in weaning pigs consuming sow milk, while other Prevotella members increased with the feeding of a solid diet. However, we discovered that taxa from the genus Fusobacterium were significantly reduced from weaning 3.6% to NP1 0.01% (Figure S1), which agrees with a previously published study [40]. In addition, Fusobacterium was reported to be dominant in preweaning pigs, and their abundance was higher in piglets with diarrhea than either healthy pigs or those receiving a practical creep-feed during the lactation stage [41]. Hence, diet type might cause the dramatic drop in Fusobacterium postweaning [42].

Besides diet type as the major influencer on gut microbiota, aging, with its physical and histological changes, could also selectively influence the microbiota. From NP1 to NP3, even with similar formulated diets, continuous internal community readjustment was observed. For instance, F6 Blautia peaked at NP1 and then experienced continuous reduction until the end of the nursery stage. F12 YRC22 slowly increased its abundance from NP1 and reached the highest abundance in NP3 (Figure 2a). These observations may have resulted from the maturation of the gut [40].

### 4.3. Lactylate and Bacillus subtilis Modulated Gut Microbiome

Gut microbiota executes multiple tasks, including food digestion, nutrient exchange, immune response, and signal transduction between the central and enteric nervous systems [43]; thus, subtle changes in the microbiota may, to some extent, lead to different physiological changes. In the current study, we provide evidence supporting how the diet intervention of lactylate and *Bacillus subtilis* modulated the gut microbiota structure. Similar to the short-term effects on ADG, pigs fed with dietary lactylate had greater microbial richness in NP1 compared to the Con group. Distinguishable microbiome communities were also observed. However, pigs that received additional *Bacillus subtilis* supplementation (LA + BM group) had the highest ADG among the four treatments but had unchanged community richness and diversity. This indicates that increased gut microbiota diversity and total observed taxa members might not be prerequisites for potential beneficial effects. The same phenomenon was observed with improved feed efficiency in pigs fed LA + BM in NP2: neither adding *Bacillus subtilis* nor a combination of lactylate and *Bacillus subtilis* increased gut community richness and diversity.

Interestingly, LEfSe analysis revealed significantly modified taxa and introduced a better understanding of how dietary interventions can enhance the health of pigs.

First, lactylate or the LA + BM combination in the diet effectively reduced the relative abundances of F16 *E. coli* in the piglets in Phase 1, which has also been validated by the culture-dependent quantification method, as previously described. *E. coli* or ETEC are major pathogens that invade during lactation and the postweaning period, causing severe dysbiosis, increased intestinal permeability, diarrhea, and reduced growth performance [23,44]. Therefore, lactylate can help protect the piglets from *E. coli* infection.

Second, supplementing lactylate to weaned pigs boosted the relative abundances of beneficial bacteria in the gut, such as F11 Prevotella, F267 Christensenellaceae, F191 Succinivibrio, and F192 Clostridiales_unclassified. These community members have been frequently discussed for their important functions in young pigs. Members of Cristensenellaceae are involved in protein- and plant-sourced fiber fermentation. In other studies, members of this family have been determined to be feed-efficiency-associated bacteria [45,46,47]. F192, an unclassified Clostridiales was stimulated in both 2.LA and 4.LA+BM groups. Clostridiales have been reported to possess multiple salutary functions. Some species can attenuate severe inflammatory responses, while others can enhance intestinal barrier functions through the production of butyrate and indolepropoinic acid that help to fuel the epithelium [48]. It is noteworthy to mention that F11 and F191 were regression-based random-forest-selected growth-performance- and feed-efficiency-associated bacterial members. F191 Succinivibrio is a core microbiota member in swine and is involved in carbohydrate metabolism, resulting in the production of acetate and succinate.

Third, lactylate expedites gut microbiota development during the first two weeks of weaning. As discussed by previous longitudinal studies, age and diet type are two major factors causing microbiota shifts [49,50]. Early gut microbiota development follows a certain specific course in healthy pigs, which is defined as microbiota maturation [28,42]. Generally, healthy pigs are less susceptible to a stressful environment and can rapidly restore their gut microbiota during the weaning to nursery transition. In our study, Prevotella gradually increased until the end of NP3 and thus, could be used as an indicator of age or gut microbiota maturation during the nursery stage. In NP1, abundant bacterial features from *Prevotella copri*, Prevotella (Figure 2a), and the genus Prevotella (Figure S1) were stimulated by dietary lactylate, which indicated a faster maturation process. Overall, our results suggest that lactylate can be used, through oral administration, to stabilize the gut microbiota community and improve growth performance.

*Bacillus subtilis* inclusion in the diet had no impact on feed intake but improved the feed conversion ratio. Hence, it significantly stimulated the efficacies of nutrient digestion and metabolism. Using regression-based random forest, we found abundances of F198 S24-7_unclassified and F141 f_Ruminococcaceae_unclassified, which were positively related to feed efficiency and were stimulated in piglets fed *Bacillus subtilis*. Similarly, taxa from Ruminococcaceae were classified as feed-efficiency-promoting members, as previously indicated [51]. On the other hand, F198 S24-7_unclassified was discovered as a predominant genus in pigs with a low feed efficiency [47]. However, another much more abundant feature, F20 S24-7 (ten times F198), demonstrated a decline in the *Bacillus subtilis* groups. F112 Coprococcus, one of the major butyrate producers, was also stimulated by *Bacillus subtilis* [52]. Overall, dietary *Bacillus* strains enriched the bacteria with high fiber fermentation efficacy, which may enhance their potential as a source of prospective DFM.

In conclusion, the current study evaluated the effects of both lactylate and *Bacillus subtilis* on growth indexes, blood cell profiles, and microbiota shifts in nursery pigs. Lactylate exhibited beneficial promoting effects on average daily gain during the first two weeks of the nursery stage. This response can be explained, in part, by the increasing levels of neutrophils in systemic circulation and the decreasing fecal *E. coli* and *ETEC* counts. The addition of lactylate in the diet increased the overall microbiome richness and maturity and modulated the gut microbiome by reducing F16 *E. coli* and increasing the beneficial bacterial features of F267 Christensenellaceae, F191 Succinivibrio, and F192 Clostridiales_unclassified. Pigs fed *Bacillus subtilis* had enriched high feed-efficiency-associated features (F141 f_Ruminococcaceae and F198 S24-7_unclassified) and butyrate-producing taxa (F122 Coprococcus). The combination of lactylate and *Bacillus subtilis* strains showed the greatest feed efficiency and increased the top-most feed-efficiency-associated taxa (F11 Prevotella) among treatments and further reduced total *E. coli* and enterotoxigenic *E. coli* counts from pigs fed lactylate alone. Collectively, these findings suggested that a synergistic effect was exerted by the lactylate and *Bacillus subtilis* mixture, and this combination could be added to diets to balance gut microbiota and improve growth performance during the early postweaning period.

## Data Availability

Data are available on the National Center for Biotechnology Information (NCBI) Short Read Archive database (SUB9202712). The BioProject accession number is PRJNA706745.

## Supplementary Materials

The following are available online at https://www.mdpi.com/article/10.3390/microorganisms9040803/s1, Figure S1: Characterization of gut microbiome compositions at the phylum and genus levels during the nursery stage. Rectal swabs were collected from all four treatments at the end of each phase and subjected to DNA extraction and 16S rDNA amplicon sequencing; Figure S2. Dietary Bacillus subtilis mixture (3.BM and 4. LA+BM) modulated the overall gut microbiome communities (alpha diversities: a and b; beta diversities: c and d) and single bacteria member (e and f) during the second phase after weaning. Dietary lactylate increased the microbial richness, and Bray-Curtis distances show a distinguishable separation between the two groups. LEfSe disclosed features with significant changes by adding the Bacillus subtilis mixture to the diet; Figure S3. Regression-based Random Forest disclosed ADG associated features (top 30) at the end of phase 2. Bacterial features F141 and F198 were positively related to FE. Adding the Bacillus subtilis mixture to the diet can enrich both features in phase 2; Table S1: Control diet composition (As fed)1; Table S2. Plasma C12 (lauric acid) and C14 (myristic acid) lactylate level (means±sem); Table S3. Main effect of adding lactylates (LA) and/or Bacillus subtilis mixture (BM) on growth performance of nursery pigs (LS means); Table S4. Lactylates and Bacillus subtilis mixture interaction effect on growth performance of nursery pigs (LS means); Table S5 lactylate and age interaction effect on peripheral complete blood cell count (LS means); Table S6 Bacillus subtilis mixture and age interaction effect on peripheral complete blood cell count (LS means); Table S7. Fecal microbial counts and prevalence obtained at baseline (Phase 0) and at the end of each nursery feed phase.
