# Supplementary material for: Effect of Lactylate and Bacillus subtilis on Growth Performance, Peripheral Blood Cell Profile, and Gut Microbiota of Nursery Pigs
Source: Microorganisms. 2021 Apr 10;9(4):803. doi: 10.3390/microorganisms9040803 (PMC8070655; doi:10.3390/microorganisms9040803)

Table S1. Control diet composition (As fed)[1].

Ingredients, %	Phase 1	Phase 2	Phase 3
Corn, Yellow Dent	44.3	55.0	47.3
Soybean meal, 48%, high protein, dehulled, sol extracted	17.6	24.3	26.5
Corn DDGS, >6 and <9% Oil	0	0	20
Poultry Fat	3	3	3
Monocalcium P	0.65	0.54	0.35
Limestone	0.48	0.79	1.13
Salt	0.25	0.50	0.50
L-Lysine	0.36	0.46	0.50
DL-Methionine	0.21	0.22	0.14
L-Threonine	0.12	0.16	0.12
L-Tryptophan	0.04	0.04	0.04
Plasma (AP-920)	3	1.5	0
Fish Meal, Menhaden	6	3	0
Milk, Whey Powder	20	10	0
L-Valine	0.09	0.11	0
L-Isoleucine	0.03	0.02	0
Milk, Lactose	3.5	0	0
Others[2]	0.433	0.433	0.433
Total	100	100	100
Calculate			
ME (kcal/kg)	3496	3461	3440
CP (%)	21.18	21.22	22.80
SID Lysine (%)	1.460	1.420	1.289
Available P (%)	0.541	0.380	0.251
Aval. P (%) with phytase	0.571	0.409	0.280
Ca (%)	0.847	0.751	0.648
SID M+C:Lys	58.0	58.0	58.0
SID Thr:Lys	60.0	60.1	60.1
SID Trp:Lys	19.1	19.1	19.0
SID Ile:Lys	55.1	55.0	60.1
SID Val:Lys	67.1	67.0	67.5
SID Leu:Lys	110.6	110.8	138.5
SID His:Lys	34.3	34.7	39.9

[1]Dietary treatments: Active lactylate (Corbion Animal Health and Nutrition, Amsterdam, Netherlands) was added at 0.2% while *Bacillus subtilis* strains (Certillus™, Arm and Hammer Animal and Food Production, Waukesha, WI) were added at 0.05%, replacing corn in the control diets to form lactylate (LA) and *Bacillus subtilis* mixture (BM) treatments. The same level of LA (0.2%) and BM (0.05%) was supplemented in diets to create lactylate + *Bacillus subtilis* mixture treatment (LA+BM). Experimental diets were fed throughout the entire trial. Diets were antibiotic-free and were formulated without pharmaceutical levels of zinc or copper.

[2]Others: 0.15% Trace mineral premix, 0.25% Vitamin premix, 0.03% Ethoxiquine, 0.003% Ronozyme HP2700 (GT). The vitamin premix provided the following per kg of complete diet: 397.5 mg of Ca as $CaCO_3$, 11,022.9 IU of vitamin A, 1,377.9 IU of vitamin D_3, 44.09 IU of vitamin E, 0.0386 mg vitamin B_{12}, 4.41 mg of menadione, 8.27 mg of riboflavin, 27.56 mg of D-pantothenic acid, and 49.6 mg of niacin. The mineral premix provided the following per kg of complete diet: 84 mg of Ca as $CaCO_3$, 165 mg of Fe as $FeSO_4$, 165 mg of Zn as $ZnSO_4$, 39.6 mg of Mn as $MnSO_4$, 16.5 mg of Cu as $CuSO_4$, 0.3 mg of I as CaI_2, and 0.3 mg of Se as Na_2SeO_3.

Table S2. Plasma C12 (lauric acid) and C14 (myristic acid) lactylate level (means±sem).

	Control	0.2% lactylate	0.2% lactylate + 0.05% *Bacillus subtilis* mixture
C12 lactylate, ng/mL			
Phase 1	5.49±0.5	119.94±25.8	146.47±37.7
Phase 2	5±0	148.54±23.5	134.02±29.3
Phase 3	5±0	138.48±20	124.68±31.6
C14 lactylate, ng/mL			
Phase 1	5±0	14.06±1.7	18.14±3.1
Phase 2	5±0	18.94±3.5	17.89±3
Phase 3	5±0	17.41±1.8	16.82±3.5

Note: Blood samples were drawn via jugular vena and collected into K2EDTA coated vacutainer tubes for plasma lactylate evaluation (Corbion R & D Laboratory, Gorinchem, Netherlands) by HPLC. The lowest detectable value was 5 ng/mL for both assays.

Table S3. Main effect of adding lactylates (LA) and/or *Bacillus subtilis* mixture (BM) on growth performance of nursery pigs (LS means).

	0% LA	0.2% LA	SEM	0% BM	0.05% BM	SEM	*P* - Value	
							LA	BM
BW, kg								
Initial	6.04	6.02	0.38	6.02	6.04	0.38	0.15	0.48
End of Phase 1	7.09	7.25	0.37	7.12	7.23	0.37	0.12	0.25
End of Phase 2	10.90	11.18	0.56	10.87	11.21	0.56	0.25	0.17
End of Phase 3	16.91	17.32	0.89	16.89	17.33	0.89	0.30	0.27
ADG, kg								
Phase 1	0.075	0.088	0.005	0.078	0.085	0.005	0.07	0.28
Phase 2	0.272	0.280	0.016	0.267	0.285	0.016	0.54	0.20
Phase 3	0.429	0.438	0.024	0.430	0.437	0.024	0.55	0.64
Phase 1&2	0.174	0.185	0.008	0.173	0.185	0.008	0.21	0.18
Overall	0.259	0.269	0.013	0.259	0.269	0.013	0.28	0.28
ADFI, kg								
Phase 1	0.157	0.171	0.005	0.168	0.160	0.005	0.01	0.18
Phase 2	0.377	0.383	0.022	0.381	0.379	0.022	0.73	0.91
Phase 3	0.608	0.646	0.036	0.618	0.636	0.036	0.14	0.49
Phase 1&2	0.267	0.277	0.012	0.274	0.270	0.012	0.29	0.62
Overall	0.380	0.400	0.020	0.389	0.392	0.020	0.16	0.84
G:F								
Phase 1	0.473	0.518	0.028	0.466	0.525	0.028	0.18	0.08
Phase 2	0.720	0.738	0.017	0.704	0.754	0.017	0.47	0.05
Phase 3	0.708	0.685	0.010	0.702	0.690	0.010	0.13	0.41
Phase 1&2	0.649	0.667	0.015	0.630	0.685	0.015	0.39	0.01
Overall	0.681	0.673	0.008	0.667	0.687	0.008	0.55	0.10

Note: Weaned pigs were randomly assigned to one of four dietary treatments with a 2 x 2 factorial arrangement including: 1) control (Con), 2) 0.2% lactylate (LA), 3) 0.05% *Bacillus subtilis* strains mixture (BM), or 4) the combination of LA and BM (LA+BM) added to the control basal diet at their respective inclusion rates in 2) and 3) to evaluate the effect of supplementing lactylate and the *Bacillus subtilis* mixture alone or in combination on body weight (BW), average daily gain (ADG), average daily feed intake (ADFI), and gain to feed ratio (G:F). Data were analyzed as a randomized complete block design in ANOVA with 2 x 2 factorial treatment arrangements in Mixed procedure of SAS (Cary, NC). The main effects were lactylate and *Bacillus subtilis* mixture. Pen served as the experimental unit. Significant difference was set at probability ≤ 0.05.

Table S4. Lactylates and *Bacillus subtilis* mixture interaction effect on growth performance of nursery pigs (LS means)

	Control	0.2% LA	0.05% BM	0.2% LA + 0.05% BM	SEM	*P* - value LA*BM
BW, kg						
Initial	6.03	6.02	6.05	6.02	0.38	0.34
End of phase 1	7.05	7.19	7.14	7.31	0.37	0.89
End of phase 2	10.82	10.92	10.98	11.45	0.59	0.46
End of phase 3	16.78	17.01	17.04	17.63	0.93	0.65
ADG, kg						
Phase 1	0.073	0.083	0.078	0.093	0.007	0.76
Phase 2	0.269	0.265	0.274	0.295	0.018	0.34
Phase 3	0.426	0.435	0.433	0.442	0.027	0.99
Phase 1&2	0.171	0.175	0.176	0.194	0.010	0.45
Overall	0.256	0.262	0.262	0.277	0.014	0.62
ADFI, kg						
Phase 1	0.154	0.181	0.159	0.161	0.007	0.03
Phase 2	0.377	0.384	0.376	0.381	0.025	0.97
Phase 3	0.600	0.636	0.615	0.657	0.041	0.91
Phase 1&2	0.266	0.283	0.268	0.271	0.014	0.49
Overall	0.377	0.401	0.384	0.400	0.022	0.80
G:F						
Phase 1	0.469	0.463	0.477	0.573	0.036	0.13
Phase 2	0.714	0.694	0.725	0.782	0.025	0.13
Phase 3	0.708	0.696	0.707	0.673	0.015	0.49
Phase 1&2	0.644	0.617	0.654	0.716	0.021	0.04
Overall	0.679	0.656	0.683	0.691	0.012	0.19

Note: Weaned pigs were randomly assigned to one of four dietary treatments with a 2 x 2 factorial arrangement including: 1) control (Con), 2) 0.2% lactylate (LA), 3) 0.05% *Bacillus subtilis* strains mixture (BM), or 4) the combination of LA and BM (LA+BM) added to the control basal diet at their respective inclusion rates in 2) and 3) to evaluate the effect of supplementing lactylate and the *Bacillus subtilis* mixture alone or in combination on body weight (BW), average daily gain (ADG), average daily feed intake (ADFI), and gain to feed ratio (G:F). Data were analyzed as a randomized complete block design in ANOVA with 2 x 2 factorial treatment arrangements in Mixed procedure of SAS (Cary, NC). The main effects were lactylate and the *Bacillus subtilis* mixture. Pen served as the experimental unit. Significant difference was set at probability ≤ 0.05.

Table S5 lactylate and age interaction effect on peripheral complete blood cell count (LS means)

	0% LA				0.2% LA					*P* - value
	d 0	d 14	d 28	d 42	d 0	d 14	d 28	d 42	SEM	LA*day
Concentration, k/μl										
WBC	9.42	16.21	17.92	16.48	9.95	18.25	17.30	15.21	0.97	0.20
Neutrophil (NE)	5.13	7.27	7.22	4.69	5.30	8.52	6.56	4.05	0.53	0.04
Lymphocyte (LY)	3.76	8.34	9.76	10.82	3.83	8.97	9.40	9.94	0.53	0.45
Monocyte (MO)	0.19	0.34	0.38	0.38	0.18	0.34	0.41	0.44	0.03	0.60
Eosinophil (EO)	0.31	0.23	0.52	0.58	0.61	0.34	0.88	0.75	0.09	0.30
Basophil (BA)	0.03	0.04	0.05	0.02	0.02	0.07	0.05	0.04	0.01	0.14
% over WBC										
Neutrophil (NEP)	52.90	44.03	40.35	28.39	50.51	46.90	36.81	26.54	1.84	0.20
Lymphocyte (LYP)	41.94	52.07	54.06	65.66	41.80	49.05	55.48	65.57	1.95	0.59
Monocyte (MOP)	2.04	2.14	2.15	2.32	1.89	1.86	2.44	2.85	0.13	< 0.01
Eosinophil (EOP)	2.87	1.52	3.11	3.51	5.60	1.86	5.01	4.81	0.50	0.02
Basophil (BAP)	0.26	0.23	0.33	0.12	0.20	0.34	0.27	0.23	0.07	0.10
Neutrophil-lymphocyte ratio (NLR)	141.64	91.03	79.95	44.95	142.01	102.01	69.72	41.51	8.29	0.41
RBC, M/μl	6.40	6.56	5.82	6.34	6.40	6.44	6.10	6.81	0.22	0.53
Hemoglobin, g/dL	8.76	8.23	7.09	8.42	9.55	8.12	8.12	9.52	0.35	0.19
Hematocrit, %	32.30	30.27	26.82	31.43	34.19	30.39	29.28	35.02	1.09	0.42

Note: Weaned pigs were randomly assigned to one of four dietary treatments with a 2 x 2 factorial arrangement including: 1) control (Con), 2) 0.2% lactylate (LA), 3) 0.05% *Bacillus subtilis* strains mixture (BM), or 4) the combination of LA and BM (LA+BM) added to the control basal diet at their respective inclusion rates in 2) and 3) to evaluate the effect of supplementing lactylate and the *Bacillus subtilis* mixture alone or in combination on peripheral blood cell count. Blood samples were drawn from a median body weight barrow from each pen via jugular vena and collected into K2EDTA coated vacutainer tubes and assayed by Hemavet 950 (Drew Scientific, Miami Lakes, FL, USA). Data were analyzed as a randomized complete block design in ANOVA with Mixed procedure of SAS (Cary, NC). The main effects were lactylate, the *Bacillus subtilis* mixture, and age of animal. Pen served as the experimental unit. Significant difference was set at probability ≤ 0.05.

Table S6 Bacillus subtilis mixture and age interaction effect on peripheral complete blood cell count (LS means)

	0% BM				0.02% BM					P - value
	d 0	d 14	d 28	d 42	d 0	d 14	d 28	d 42	SEM	BM*age
Concentration, k/µl										
WBC	9.94	18.06	18.82	14.40	9.42	16.40	16.40	17.30	0.97	< 0.01
Neutrophil (NE)	5.25	8.49	7.35	4.09	5.18	7.30	6.42	4.64	0.53	0.06
Lymphocyte (LY)	4.00	8.82	10.30	9.28	3.59	8.50	8.86	11.47	0.53	< 0.01
Monocyte (MO)	0.20	0.35	0.38	0.38	0.18	0.33	0.40	0.44	0.03	0.36
Eosinophil (EO)	0.46	0.34	0.73	0.62	0.46	0.23	0.67	0.71	0.09	0.25
Basophil (BA)	0.03	0.07	0.05	0.03	0.02	0.04	0.05	0.03	0.01	0.27
% over WBC										
Neutrophil (NEP)	51.10	47.06	38.46	28.12	52.31	43.87	38.70	26.81	1.84	0.67
Lymphocyte(LYP)	42.32	48.80	55.25	64.74	41.43	52.32	54.29	66.49	1.95	0.57
Monocyte (MOP)	1.97	1.92	2.07	2.64	1.96	2.07	2.52	2.53	0.13	0.14
Eosinophil (EOP)	4.35	1.85	3.96	4.33	4.13	1.53	4.16	3.99	0.50	0.95
Basophil (BAP)	0.28	0.36	0.26	0.18	0.19	0.21	0.34	0.17	0.07	0.32
Neutrophil-lymphocyte ratio (NLR)	135.07	104.16	73.72	44.53	148.57	88.89	75.96	41.93	8.29	0.48
RBC, M/µl	6.65	6.39	5.79	6.86	6.15	6.61	6.13	6.29	0.22	0.07
Hemoglobin, g/dL	9.45	8.23	7.69	9.54	8.86	8.12	7.52	8.40	0.35	0.34
Hematocrit, %	34.69	30.40	27.71	35.02	31.80	30.25	28.39	31.43	1.09	0.10

Note: Weaned pigs were randomly assigned to one of four dietary treatments with a 2 x 2 factorial arrangement including: 1) control (Con), 2) 0.2% lactylate (LA), 3) 0.05% *Bacillus subtilis* strains mixture (BM), or 4) the combination of LA and BM (LA+BM) added to the control basal diet at their respective inclusion rates in 2) and 3) to evaluate the effect of supplementing lactylate and the *Bacillus subtilis* mixture alone or in combination on peripheral blood cell count. Blood samples were drawn from a median body weight barrow from each pen via jugular vena and collected into K2EDTA coated vacutainer tubes and assayed by Hemavet 950 (Drew Scientific, Miami Lakes, FL, USA). Data were analyzed as a randomized complete block design in ANOVA within Mixed procedure of SAS (Cary, NC). The main effects were lactylate, the *Bacillus subtilis* mixture, and age of animal. Pen served as the experimental unit. Significant difference was set at probability ≤ 0.05.

Table S7. Fecal microbial counts and prevalence obtained at baseline (Phase 0) and at the end of each nursery feed phase.

						P - value		
$\log_{10}$ CFU/g	Control	BM	LA	BM + LA	SE	BM	LA	BM*LA
Phase 0-Initial								
Total Clost	6.17	6.29	6.75	6.51	0.44	0.886	0.34	0.67
CpA	6.15	6.28	6.70	6.43	0.44	0.874	0.40	0.63
Total *E. coli*	6.71	7.09	7.11	6.45	0.47	0.753	0.79	0.25
ETEC	N/D	N/D	N/D	N/D	-	-	-	-
Total Strep	6.50	6.06	7.36	6.59	0.36	0.087	0.05	0.63
S. suis	5.94	5.79	6.74	5.25	0.35	0.018	0.69	0.05
Phase 1								
Total Clost	3.99	3.94	3.84	3.90	0.21	0.975	0.64	0.80
CpA	3.77	3.94	3.84	3.90	0.18	0.502	0.92	0.73
Total *E. coli*	7.77	7.67	7.04	6.20	0.44	0.264	0.01	0.38
ETEC	7.75	7.66	7.04	6.20	0.44	0.276	0.01	0.37
Total Strep	8.43	8.08	8.54	8.57	0.20	0.405	0.12	0.33
S. suis	5.02	4.91	4.96	4.70	0.25	0.445	0.56	0.76
Phase 2								
Total Clost	3.73	3.75	3.70	3.96	0.15	0.336	0.52	0.39
CpA	3.73	3.75	3.70	3.96	0.15	0.337	0.52	0.39
Total *E. coli*	6.27	6.65	6.32	6.07	0.35	0.844	0.44	0.35
ETEC	4.97	5.94	5.40	5.47	0.46	0.248	0.96	0.31
Total Strep	8.36	8.66	8.85	8.61	0.17	0.877	0.19	0.12
S. suis	5.72	4.99	5.06	4.70	0.34	0.110	0.16	0.58
Phase 3								
Total Clost	5.13	5.08	6.18	6.45	0.34	0.723	< 0.01	0.63
CpA	5.03	5.06	5.79	6.07	0.39	0.683	0.02	0.74
Total *E. coli*	5.55	5.87	5.34	5.29	0.37	0.709	0.27	0.60
ETEC	4.80	5.51	5.25	4.83	0.37	0.695	0.74	0.12
Total Strep	7.08	6.10	6.78	7.03	0.34	0.278	0.35	0.07
S. suis	5.13	4.70	4.8	5.00	0.20	0.561	0.94	0.11

Note: Weaned pigs were randomly assigned to one of four dietary treatments with a 2 x 2 factorial arrangement including: 1) control (Con), 2) 0.2% lactylate (LA), 3) 0.05% *Bacillus subtilis* strains mixture (BM), or 4) the combination of LA and BM (LA+BM) added to the control basal diet at their respective inclusion rates in 2) and 3) to evaluate the effect of supplementing lactylate and *Bacillus subtilis* mixture alone or in combination on fecal microbial count. Fresh fecal samples from a median body weight barrow were collected at the end of phase 1, 2, and 3 to determine total *Clostridium*, *Clostridium perfringens* A, total *E. coli*, and enterotoxigenic *E. coli* count.

Figure S1. Characterization of gut microbiome compositions at the phylum and genus levels during the nursery stage. Rectal swabs were collected from all four treatments at the end of each phase and subjected to DNA extraction and 16S rDNA amplicon sequencing.

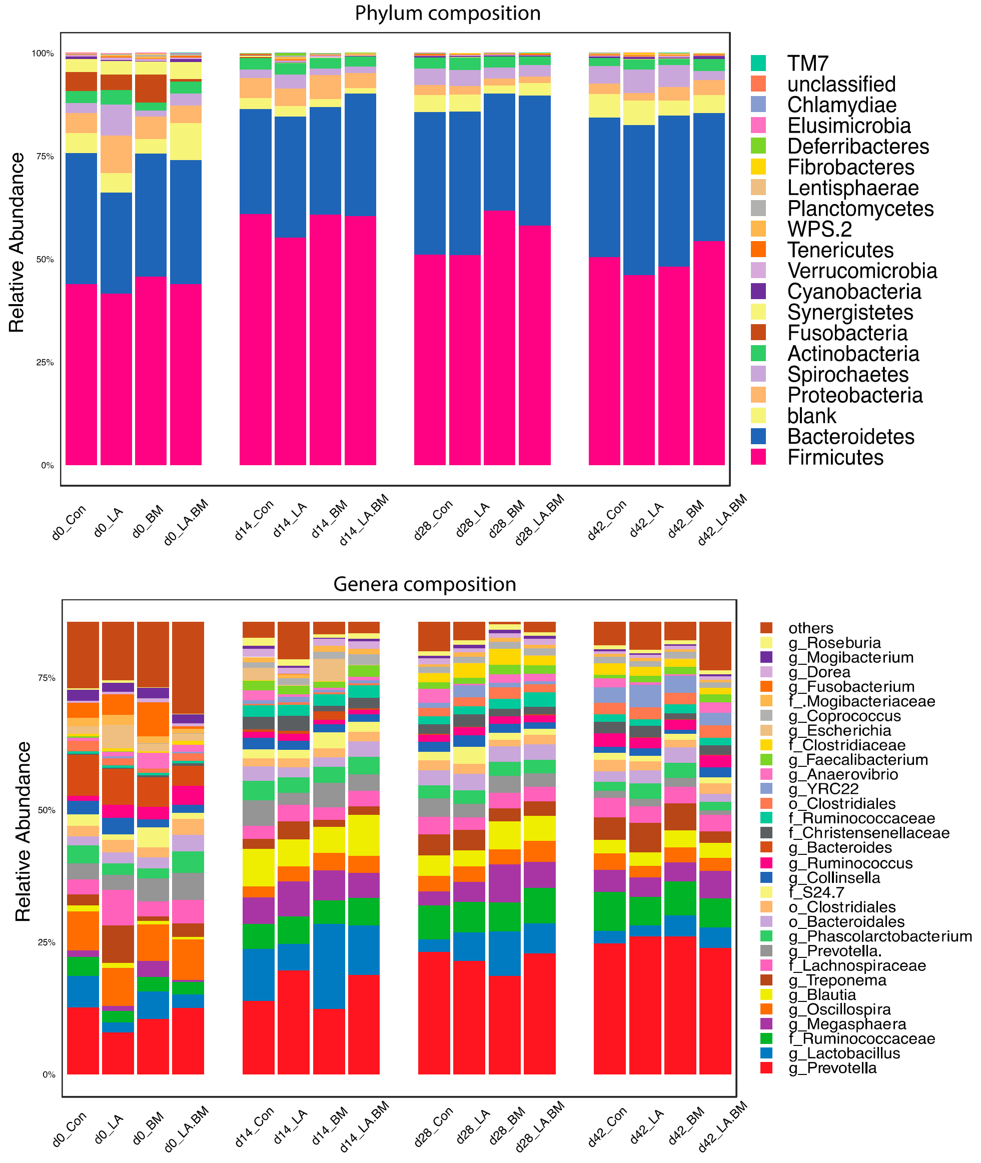

Figure S2. Dietary *Bacillus subtilis* mixture (3.BM and 4. LA+BM) modulated the overall gut microbiome communities (alpha diversities: a and b; beta diversities: c and d) and single bacteria member (e and f) during the second phase after weaning. Dietary lactylate increased the microbial richness, and Bray-Curtis distances show a distinguishable separation between the two groups. LEfse disclosed features with significant changes by adding the *Bacillus subtilis* mixture to the diet.

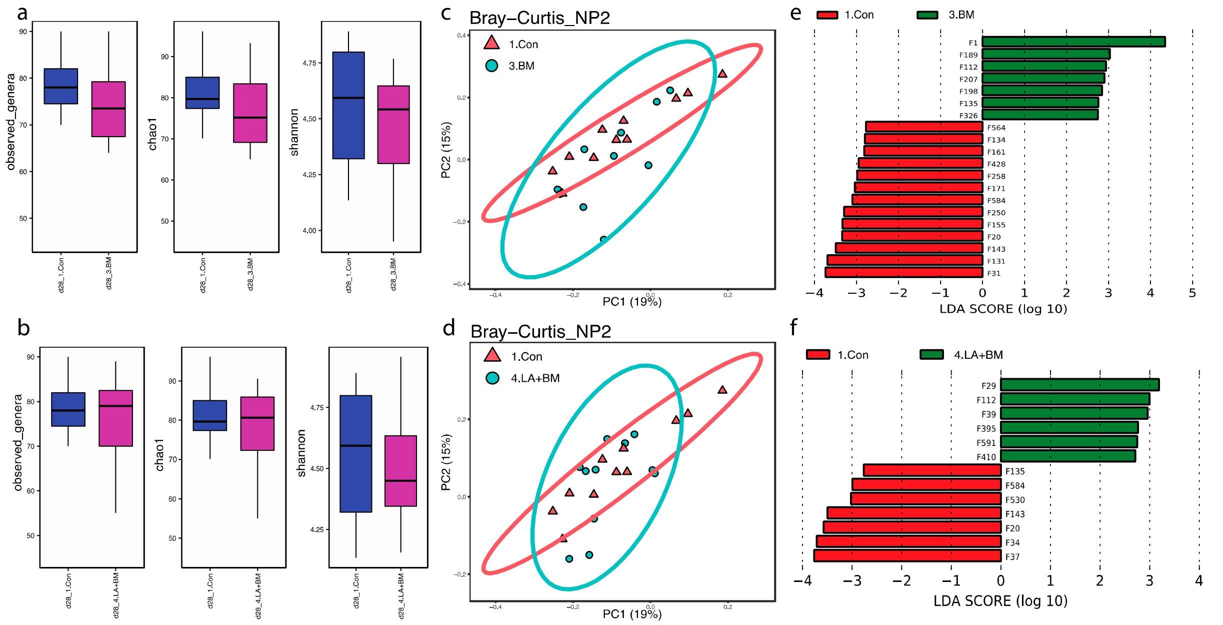

Figure S3. Regression-based Random Forest disclosed ADG associated features (top 30) at the end of phase 2. Bacterial features F141 and F198 were positively related to FE. Adding the *Bacillus subtilis* mixture to the diet can enrich both features in phase 2.

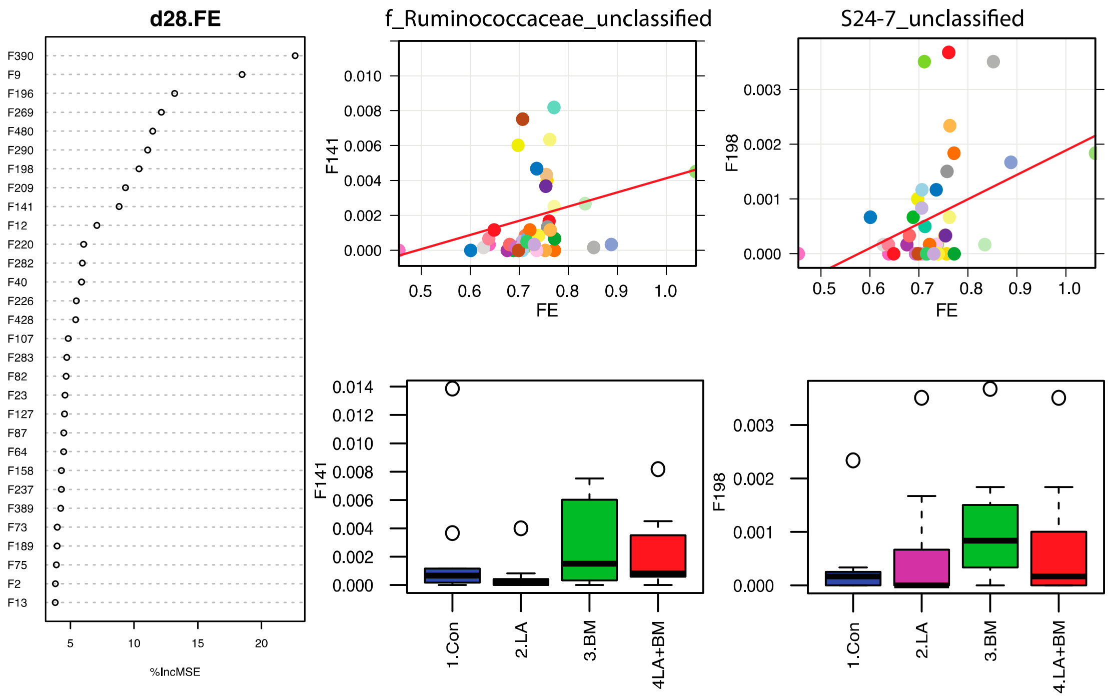

Supplement: Supplementary file 1 [file microorganisms-09-00803-s001.pdf]
